# New HTLV-1 and HTLV-2 infections in rural population, in North of Gabon, Central Africa

**DOI:** 10.1186/1742-4690-10-S1-P110

**Published:** 2013-10-11

**Authors:** Augustin Mouinga Ondeme, Rodrigue Bikangui, Ulrick Bisvigou, Paul Ngari, François Rouet

**Affiliations:** 1 Laboratoire de Rétrovirologie, Centre International de Recherches Médicales de Franceville (CIRMF), Franceville, Gabon; 2 Unité de Recherche et d’Analyses Médicales, CIRMF, Franceville, Gabon

## Background

The emergence of Human retroviruses, in Central Africa, may occur in contact with non human primates, during hunting and butchering. Recent studies described several cases of human infections with Human T-cell Lymphotropic Virus (HTLV) as the result of simian zoonotic transmissions, in south Cameroon corresponding to north Gabon.

To search for new HTLV infections (HTLV-1, HTLV-2) and to investigate zoonotic infection, we conducted a study among individuals hunting and butchering of wild non-human primates, in rural area in north of Gabon, Central Africa.

## Materials and methods

This study was conducted during 2 weeks in May 2013, in rural villages in Gabon. We studied 222 people, with 15years and up, from the general adult population (mean age 55 years, 61 women and 162 men) experiencing hunting or butchering. Blood was collected in EDTA tube, and HTLV-1/2 immunoenzymatic test (ELISA) was performed as a screening test. Because of the unavailability of the Western Blot tests during the study, Positive samples were confirmed and discriminated by PCR in *tax-rex* region with specific primers. Phylogenetic analyses using Neighbour-Joining were done.

## Results

Among a total of 222 persons, seropositivity in HTLV was found in 18 (8.10%), 5 (2.25%) were women and 13 (5.85%) men. Men were hunters while women recognized butchering activities. PCR of 18 samples showed that 5 were HTLV-1 positive (2 men and 3 women); one HTLV-2, isolated from a hunter; 12 were not amplified (5 of them showed a high optic density by serology). Phylogenetic analysis confirmed PCR results obtained for HTLV-1 and HTLV-2.

## Conclusions

Our results show HTLV-1 and HTLV-2 Infections in a risk group in north of Gabon, corresponding to south of Cameroon, Central Africa. These results suggest a probable simian origin of HTLV-1 and HTLV-2 described subtypes. Further studies are needed to best understand the interspecies transmission of these complex retroviruses; and to explore the intra familial transmission.

